# Multiplex PCR-Based Nanopore Sequencing and Epidemiological Surveillance of *Hantaan orthohantavirus* in *Apodemus agrarius*, Republic of Korea

**DOI:** 10.3390/v13050847

**Published:** 2021-05-06

**Authors:** Kyungmin Park, Seung-Ho Lee, Jongwoo Kim, Jingyeong Lee, Geum-Young Lee, Seungchan Cho, Seung Ho Lee, Kkothanahreum Park, Jin Sun No, Shailesh Budhathoki, Yu-Jin Kim, Young-Su Kim, Heung-Chul Kim, Terry A. Klein, Won-Keun Kim, Jin-Won Song

**Affiliations:** 1 BK21 Graduate Program, Department of Biomedical Sciences, Korea University College of Medicine, Seoul 02841, Korea; kmpark0131@korea.ac.kr (K.P.); hotdog442@korea.ac.kr (J.K.); 2 Department of Microbiology, Korea University College of Medicine, Seoul 02841, Korea; leeds1104@korea.ac.kr (S.-H.L.); yoj0702@korea.ac.kr (J.L.); gemyeng002@korea.ac.kr (G.-Y.L.); schanchan@korea.ac.kr (S.C.); meales@korea.ac.kr (S.H.L.); pkhar@korea.ac.kr (K.P.); 3 Division of High-Risk Pathogens, Bureau of Infectious Diseases Diagnosis Control, Korea Disease Control and Prevention Agency, Cheongju 28726, Korea; njs2564@gmail.com; 4 Department of Microbiology, College of Medicine, Hallym University, Chuncheon 24252, Korea; shailesh.sas24@gmail.com (S.B.); wkkim1061@hallym.ac.kr (W.-K.K.); 5 Department of Preventive Medicine, Army Headquarters, Gyeryong 32800, Korea; rok1632@army.mil.kr; 6 Gangwon Institute of Health and Environment, Infectious Disease Research Department, Chuncheon 24203, Korea; gys@korea.kr; 7 Force Health Protection and Preventive Medicine, MEDDAC-Korea, 65th Medical Brigade, Unit 15281, APO AP 96271-5281, USA; hungchol.kim2.ln@mail.mil (H.-C.K.); terry.a.klein2.civ@mail.mil (T.A.K.); 8 Institute of Medical Research, College of Medicine, Hallym University, Chuncheon 24252, Korea

**Keywords:** Hantaan virus (HTNV), hemorrhagic fever with renal syndrome (HFRS), next-generation sequencing (NGS), whole-genome sequencing, amplicon-based NGS, MinION sequencing, phylogenetic analysis, genetic diversity, viral genome surveillance

## Abstract

Whole-genome sequencing of infectious agents enables the identification and characterization of emerging viruses. The MinION device is a portable sequencer that allows real-time sequencing in fields or hospitals. *Hantaan orthohantavirus* (Hantaan virus, HTNV), harbored by *Apodemus agrarius*, causes hemorrhagic fever with renal syndrome (HFRS) and poses a critical public health threat worldwide. In this study, we aimed to evaluate the feasibility of using nanopore sequencing for whole-genome sequencing of HTNV from samples having different viral copy numbers. Amplicon-based next-generation sequencing was performed in *A. agrarius* lung tissues collected from the Republic of Korea. Genomic sequences of HTNV were analyzed based on the viral RNA copy numbers. Amplicon-based nanopore sequencing provided nearly full-length genomic sequences of HTNV and showed sufficient read depth for phylogenetic analysis after 8 h of sequencing. The average identity of the HTNV genome sequences for the nanopore sequencer compared to those of generated from Illumina MiSeq revealed 99.8% (L and M segments) and 99.7% (S segment) identities, respectively. This study highlights the potential of the portable nanopore sequencer for rapid generation of accurate genomic sequences of HTNV for quicker decision making in point-of-care testing of HFRS patients during a hantavirus outbreak.

## 1. Introduction

Orthohantaviruses (family, *Hantaviridae*; order, *Bunyavirales*) are enveloped negative-sense single-stranded RNA viruses [1]. The viral genome consists of tripartite RNA segments (large, medium, and small) encoding an RNA-dependent RNA polymerase (RdRp), two surface glycoproteins (Gn and Gc), and a nucleocapsid (N) protein, respectively [2]. Orthohantaviruses are the etiological agents of hemorrhagic fever with renal syndrome (HFRS), with case fatality rates varying from <1% to 15% in Eurasia [3]. HFRS is mainly caused by Hantaan virus (HTNV), Seoul virus, Dobrava virus, and Puumala virus [4]. The natural reservoir hosts of hantaviruses include rodents (Rodentia), bats (Chiroptera), and insectivores (Soricomorpha) [5,6,7]. Hantaviruses are transmitted to humans when aerosolized infectious particles from the saliva, urine, and feces of infected rodents are inhaled through the respiratory tract [8,9].

Next-generation sequencing (NGS) technologies have been applied to a wide range of fields in virology, including metagenomics of the virome, whole-genome sequencing for population-level surveillance, the tracking of the spread of infectious agents, the development of vaccines, and the identification of putative pathogens [10,11,12,13]. Whole-genome sequencing plays a critical role in understanding the characteristics and transmission dynamics during the outbreaks of emerging or re-emerging viruses that pose a public health threat [14,15,16,17]. However, a significant limitation of whole-genome sequencing for the identification of viral sequences is the ultra-low copy number of target genomes in clinical or environmental sources. To detect low-titer viruses, researchers have developed various NGS approaches, including sequence-independent, single-primer amplification (SISPA); specific oligonucleotide probe-mediated enrichment of target viral nucleic acids; amplicon-based NGS; and small-RNA deep sequencing [18,19,20,21].

MinION (Oxford Nanopore Technologies) is a palm-sized portable sequencer that is smaller and cheaper than conventional sequencing platforms; it allows real-time sequencing in field situations or hospitals [22,23]. The nanopore sequencing has allowed researchers to reveal the dynamic genetic diversity and geographic distribution of the rabies virus in field [24]. Previous studies have used nanopore sequencing in point-of-care testing for the real-time identification of infectious agents, including severe acute respiratory syndrome coronavirus 2, Chikungunya virus (CHIKV), Ebola virus (EBOV), Zika virus (ZIKV), and hepatitis C virus in clinical samples [25,26,27]. However, to our knowledge, nanopore-based NGS has not been used to conduct whole-genome sequencing of orthohantaviruses directly from the lung tissue of a natural host, *Apodemus agrarius* (the striped field mouse).

In this study, we evaluated whether the MinION device could be used for the whole-genome sequencing of HTNV independently of virus culture and determined whether it could rapidly and accurately generate genomic sequences for the identification of genetic diversity at the variants. The epidemiological surveillance of small mammals showed the genetic diversity and geographical prevalence of HTNV in HFRS-endemic areas in the Republic of Korea (ROK). This study provides important insights into the application of nanopore sequencing for genomic-based diagnosis and analysis of orthohantaviruses from clinical and animal specimens in the field.

## 2. Materials and Methods

### 2.1. Ethics Statement

Rodents and shrews were handled in accordance with the ethical guidelines of the Korea University Institutional Animal Care and Use Committee (KUIACUC #2016-49). Small mammals were euthanized via cardiac puncture under isoflurane anesthesia. Necropsy and experiments were performed in a biosafety level 3 laboratory at Korea University.

### 2.2. Sample Collection

Field trappings were performed using Sherman live traps (8 cm × 9 cm × 23 cm; H. B. Sherman, Tallahassee, FL, USA) in Gyeonggi and Gangwon Provinces, ROK, in 2019. For each day, a total of 100 traps was placed in unmanaged grasses, herbaceous vegetations, farmlands, mountains, fields, and forests at intervals of 1–5 m for 1–3 days. The captured rodents and shrews were sequentially numbered, placed in secure containers, and transported to the biosafety level 3 facility at Korea University in accordance with the safety guidelines. Sera and multiple tissue samples were aseptically obtained from the small mammals. The sera and heart fluids were separated by centrifugation for 5 min at 4 °C. The tissue samples were stored at −80 °C until use.

### 2.3. Mitochondrial DNA Analysis

Total DNA was extracted from the liver tissues using a High Pure PCR Template Preparation Kit (Roche, Basel, Switzerland). Mitochondrial cytochrome *b* (Cyt *b*) gene-specific PCR was performed using universal primers forward: 5′-CGA AGC TTG ATA TGA AAA ACC ATC GTT G-3′ and reverse: 5′-CTG GTT TAC AAG ACC AGA GTA AT-3′ [28]. Polymerase chain reaction (PCR) was performed in 25 μL reaction mixtures, containing 2.5 mM dNTP (ELPIS Biotech, Daejeon, South Korea) and 1 U of Super-Therm Taq polymerase (JMR Holdings, London, UK). First cycling conditions consisted of an initial denaturation at 95 °C for 5 min followed by 8 cycles with denaturation at 94 °C for 1 min, annealing at 50 °C for 1 min, and elongation at 72 °C for 2 min. Second cycling was conducted by 30 cycles of denaturation at 94 °C for 1 min, annealing at 55 °C for 1 min, and elongation at 72 °C for 2 min, and final cycle at 72 °C for 10 min in a thermal cycler (ProFlex PCR System, Life Technology, CA, USA). The obtained sequences were deposited in GenBank (accession numbers: MW796110–MW796121).

### 2.4. Indirect Immunofluorescence Antibody Test

The sera or heart fluids were diluted 1:32 and 1:2, respectively, and placed in duplicate wells of acetone-fixed Vero E6 cells infected with HTNV. The plate was incubated at 37 °C for 30 min, then the cells were washed with Phosphate-Buffered Saline (PBS) and distilled water (D.W.). The slides were treated with fluorescein isothiocyanate-conjugated anti-mouse (for rodents) or mouse/rat (for shrews) immunoglobulin G (IgG) antibodies (MP Bio, CA, USA). Subsequently, the slides were incubated at 37 °C for 30 min, washed with PBS and D.W., and examined under a fluorescence microscope (Axio Scope; Zeiss, Berlin, Germany). Virus-specific fluorescence is indicative of HTNV infection.

### 2.5. Reverse Transcription-Polymerase Chain Reaction

Total RNA was extracted from the lung tissues of the rodents using TRI Reagent Solution (Ambion, Austin, Texas, USA) according to the manufacturer’s instructions. cDNA was synthesized from 1 µg of total RNA by using the High Capacity RNA-to-cDNA kit (Applied Biosystems, Foster City, CA, USA) with a random hexamer and OSM55 (5′-TAG TAG ACT CC-3′). PCR conditions and primer sequences for amplification of HTNV genomes have been previously described [29]. Oligonucleotide primer sequences followed up Han-L-F1 (outer): 5′-ATG TAY GTB AGT GCW GAT GC-3′ and Han-L-R1 (outer): 5′-AAC CAD TCW GTY CCR TCA TC-3′, Han-L-F2 (inner): 5′-TGC WGA TGC HAC IAA RTG GTC-3′ and Han-L-R2 (inner): 5′-GCR TCR TCW GAR TGR TGD GCA A-3′ for the L segment; G2F1 (outer): 5′-TGG GTG CAA GTG C-3′, G2-2 (outer): 5′-ACA TGC TGT ACA GCC TGT GCC-3′, G2-1 (inner): 5′-TGG GCT GCA AGT GCA TCA GAG-3′, G2-4 (inner): 5′-ATG GAT TAC AAC CCC AGC TCG-3′ for the M segment; HTN-S6 (outer): 5′-AGC TCI GGA TCC ATI TCA TC-3′ and OSQ84 (outer): 5′-ATC TTA CAT CCT TTG TCG TCC C-3′, HTN-S4 (inner): 5′-GAI IGI TGT CCA CCA ACA TG-3′ and OSQ85 (inner): 5′-AGT TGT CCA CAG CCT CCT TT-3′ for the S segment. First- and second-round PCR were performed in 25 μL reaction mixture containing 2.5 mM dNTP (ELPIS Biotech, Daejeon, South Korea), 1U of Super-Therm Taq DNA polymerase (JMR Holdings, London, UK), and 1.5 μg of cDNA with 10 pM of each primer. Cycling condition consisted of initial denaturation at 94 °C for 5 min, followed by 6 cycles of denaturation at 94 °C for 30 sec, annealing at 37 °C for 40 sec, and elongation at 72 °C for 1 min. Second cycling was performed by 32 cycles of denaturation at 94 °C for 30 sec, annealing at 42 °C for 40 sec, and elongation at 72 °C for 1 min, and final cycle at 72 °C for 5 min (ProFlex PCR System, Life Technology, CA, USA). PCR products were purified using MinElute PCR purification kit (Qiagen, Hilden, Germany). Amplicons were sequenced in forward and reversed directions of each PCR product using BigDye Terminator v3.1 Cycle Sequencing Kit (Applied Biosystems) on automated sequencer (ABI 3730XL DNA Analyzer, Applied Biosystems).

### 2.6. Quantitative PCR

Quantitative PCR (qPCR) was performed using SYBR Green PCR Master Mix (Applied Biosystems, Foster City, CA, USA) on a Quantstudio 5 Flex Real-Time PCR System (Applied Biosystems, Foster City, CA, USA) using the following reaction steps: denaturation at 95 °C for 10 min, followed by 40 cycles at 95 °C for 15 s and 60 °C for 1 min. The forward (5′-TTA TTG TGC TCT TCA TGG TTG C-3′) and reverse primer (5′-CAT CCC CTA AGT GGA AGT TGT C-3′) sequences of the HTNV S segment were used [30]. The viral copy number of the HTNV genomes was calculated using a linear regression curve described in a previous study [20].

### 2.7. Multiplex PCR-Based Enrichment

cDNA was amplified using HTNV-specific primer mixtures using Solg 2X Uh-Taq PCR Smart Mix (Solgent, Daejeon, Republic of Korea) according to the manufacturer’s instructions. The PCR conditions and primer information were described in a previous study [18]. The enrichment was performed in 25 μL reaction mixture containing 12.5 μL 2X Uh premix, 2.0 μL of each primer mixture, 10.5 μL of D.W., and 1.0 μL of DNA template. Multiplex PCR was performed as follows: a cycle at 95 °C for 15 min, followed by 40 cycles or 25 cycles at 95 °C for 20 s, 50 °C for 40 s, and 72 °C for 1 min, and a cycle at 72 °C for 3 min.

### 2.8. Library Preparation for Sequencing on MinION Platform

**Figure 1:**
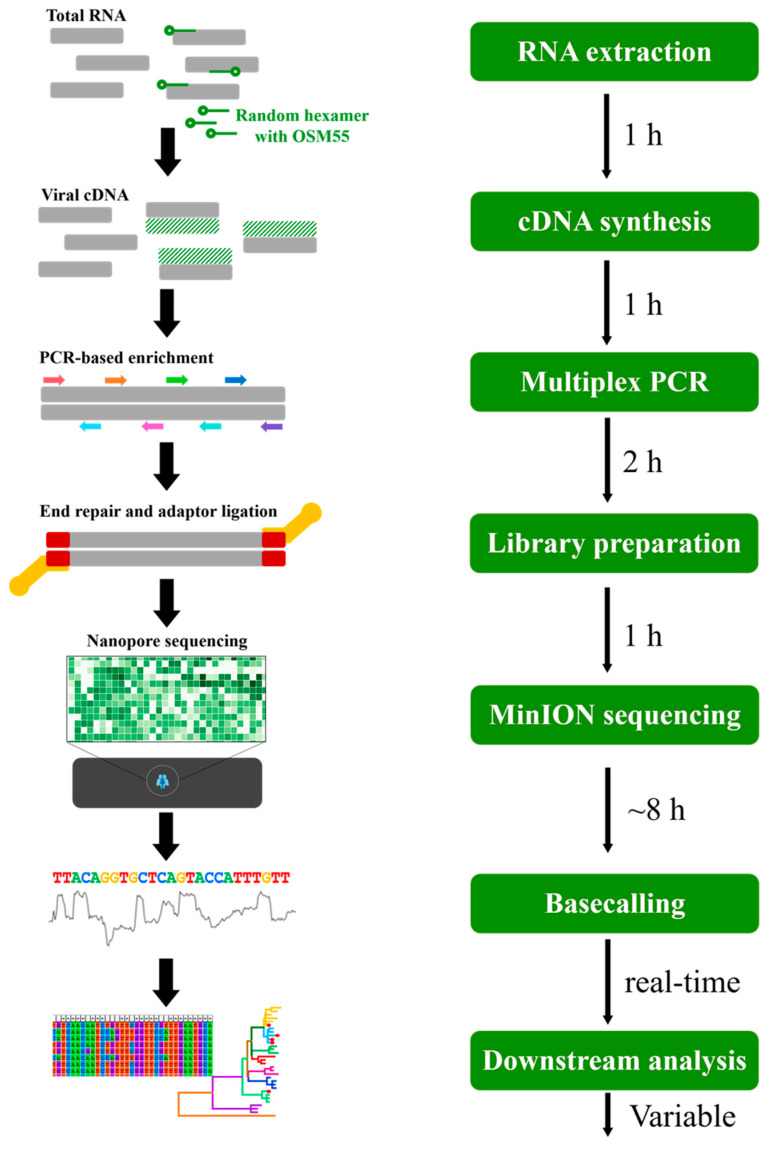
Workflow overview of the amplicon-based nanopore sequencing for Hantaan virus (HTNV) used in this study. Total RNA was extracted from rodent lung tissues, and cDNA was synthesized from 1 µg of total RNA with a random hexamer and OSM55. cDNA was amplified using HTNV-specific primer mixtures for library preparation. Barcoded libraries were pooled, ligated to sequencing adapter, and sequenced using the MinION device with FLO-MIN106 (R9.4) flow cell for 8 h. The basecalled sequences were combined into a single FASTA file, and the reads were mapped to the reference genome sequences. The consensus sequences were extracted for downstream analysis.

The library was prepared using Ligation Sequencing Kit (SQK-LSK109) and Native Barcoding Kit (EXP-NBD104 and NBD 114) according to the standard protocols (Oxford Nanopore Technologies, London, UK). Amplified libraries were purified using AMPure XP beads (Beckman Coulter, CA, USA). Barcoded libraries were pooled, ligated to sequencing adapters, and sequenced using the MinION by using FLO-MIN106 (R9.4) flow cell for 16 h. Real-time basecalling was performed using Guppy (v3.0.3; MinKNOW software) in the MK1C device. Raw data were demultiplexed, and the adaptor sequences were trimmed. Only reads with a default Q-score of 7 were included in the subsequent analysis to improve the reliability of the data. The filtered sequences were combined into a single FASTA file for each barcode by using Porechop (v.9.0). The reads were mapped to the reference genome sequence of HTNV 76-118, and the consensus sequences were extracted using the CLC Genomics Workbench (v7.5.2; Qiagen, Hilden, Germany) [20]. The consensus sequences were manually polished using the error-correction method. An indel, insertion, or deletion in the open reading frame (ORF) was considered as a nanopore sequencing error. If the indels occurred only in the consensus sequence, the correction was performed based on the reference sequences. The uncovered sequences of HTNV tripartite genomes were determined using conventional nested RT-PCR. The workflow and time-span overview of the nanopore sequencing used in this study are shown in Figure 1. The genomic sequences of HTNV are described in Table S1.

### 2.9. Library Preparation for Sequencing on MiSeq Platform

DNA libraries were prepared using the TruSeq Nano DNA LT sample preparation kit (Illumina, San Diego, USA) according to the manufacturer’s instructions and a previously described method [18]. NGS was performed on a MiSeq benchtop sequencer platform (Illumina, San Diego, USA) with 2 × 150 bp by using a MiSeq reagent kit v2 (Illumina, San Diego, USA). Total reads were trimmed to the adaptor and index sequences, and low-quality reads were filtered using CLC Genomics Workbench version 7.5.2 (CLC Bio, Cambridge, MA, USA). The filtered reads were mapped to the tripartite genome sequence of HTNV 76-118, and consensus sequences were extracted. The uncovered genomic sequences of HTNV L, M, and S segments were determined by conventional nested RT-PCR. The genomic sequences of HTNV are described in Table S1.

### 2.10. Phylogenetic Analysis

The genomic sequences of the HTNV L, M, and S segments were aligned using the Clustal W algorithm in Lasergene version 5 (DNASTAR Inc., Madison, WI, USA). Phylogenetic analysis was performed using the best-fit TN93+G+I (for L segment) and T92+G+I (for M and S segments) substitution models of evolution by using the maximum-likelihood (ML) method in MEGA 7.0 [31]. Topologies were evaluated using bootstrap analysis of 1000 iterations.

## 3. Results

### 3.1. Trapping Sites of Small Mammals from the ROK

**Figure 2:**
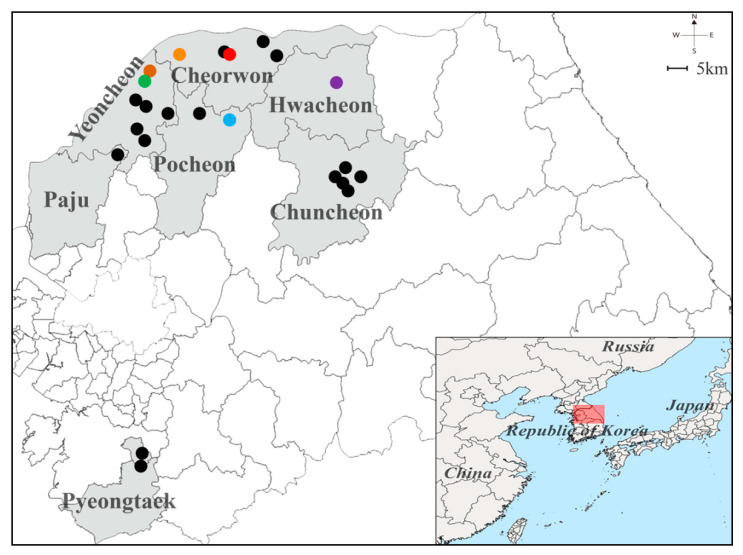
Geographical location of the trapping sites of Hantaan virus (HTNV) collected from Gyeonggi and Gangwon Provinces, the Republic of Korea. The geographic map shows different trapping areas where small mammals were captured at Gyeonggi and Gangwon Provinces in 2019. The colored circles indicate the HTNV RNA positive sites: Pocheon, blue (Jangam-ri); Yeoncheon, green (Dosin-ri) and brown (Sang-ri); Cheorwon, orange (Gwanu-ri) and red (Dochang-ri); Hwacheon-gun, violet (Pungsan-ri). The black circles represent the locations where no HTNV RNA was detected: Paju (Jeogam-ri); Pocheon (Jail-ri); Yeoncheon (Hyunga-ri, Dongi-ri, Ohkye-ri, Yangwon-ri, and Bugok-ri); Cheorwon (Eupnae-ri, Munhye-ri, and Chungyang-ri); and Chuncheon (Gamjeong-ri, Sinchon-ri, Jinae-ri, Balsan-ri, and Yulmun-ri). A Quantum Geographical Information System (QGIS) 3.10 for Mac was used to create the map, which was modified in Adobe Illustrator CC 2019.

Small mammals were collected from multiple sites, including Paju, Pocheon, Pyeongtaek, Yeoncheon, Cheorwon, Chuncheon, and Hwacheon, in Gyeonggi and Gangwon Provinces, ROK, in 2019 (Figure 2). Geographic information for the sites is shown in Table S2. The captured animals included 303 *A. agrarius*, 13 *Crocidura lasiura,* 3 *C. shantungensis*, 17 *Mus musculus*, four *Myodes regulus*, 5 *Micromys minutus*, 1 *Microtus fortis*, 3 *Rattus norvegicus,* and 1 *Tscherskia triton* (Table 1). *A. agrarius* (86.6% of all individuals) was the most frequently collected species at all trapping sites. Phylogenetic analysis of the mitochondrial Cyt *b* gene showed that the captured rodents formed a genetic lineage with *A. agrarius* (Figure S1).

### 3.2. Serological and Molecular Prevalence of HTNV Collected from the ROK

A total of 20/303 (6.6%) rodents were seropositive, of which 1/18 (5.6%), 5/48 (10.4%), 7/105 (6.7%), 1/38 (2.6%), and 6/46 (13.0%) were detected in Pocheon, Yeoncheon, Cheorwon, Chuncheon, and Hwacheon, respectively; no seropositive rodents were detected in Paju and Pyeongtaek (Table 2). The seropositive samples were examined for hantaviral RNA by using HTNV-specific RT-PCR. In total, 12/20 (60%) seropositive *A. agrarius* harbored HTNV RNA, including 1/1 (100%) in Pocheon, 2/5 (40%) in Yeoncheon, 6/7 (85.7%) in Cheorwon, and 3/6 (50%) in Hwacheon. Hantaviral antibodies were not detected in the captured rodents and shrews except for *A. agrarius*.

### 3.3. HTNV RNA Loads and Determination of Viral Copy Number from A. agrarius Lung Tissues

Viral RNA load was measured in the lung tissues of the captured rodents (Aa19-36, Aa19-38, Aa19-39, Aa19-57, Aa19-89, Aa19-144, Aa19-152, Aa19-153, Aa19-167, Aa19-233, Aa19-236, and Aa19-278; Table 3). Aa19-233 and Aa19-278 harbored the highest amount of HTNV RNA (Ct value, 20.5–22.2), corresponding to 10^4^ to 10^5^ copies/μL in the evaluated rodents. Aa19-36 and Aa19-236 showed Ct values ranging from 29.4 to 29.7 (10^2^ to 10^3^ copies/μL of viral RNA). The Ct values of the rodents Aa19-38, Aa19-57, Aa19-89, Aa19-153, and Aa19-167 ranged from 31.8 to 34.5, indicating that the samples contained 10^1^ to 10^2^ copies/μL of viral RNA. Aa19-144 and Aa19-152 consisted of 1 to 10^1^ copies/μL of HTNV RNA with Ct values ranging from 36.4 to 37.3. In Aa19-39, HTNV RNA was not detectable by qPCR.

### 3.4. Multiplex PCR-Based NGS of HTNV Using MinION or MiSeq Platforms

**Figure 3:**
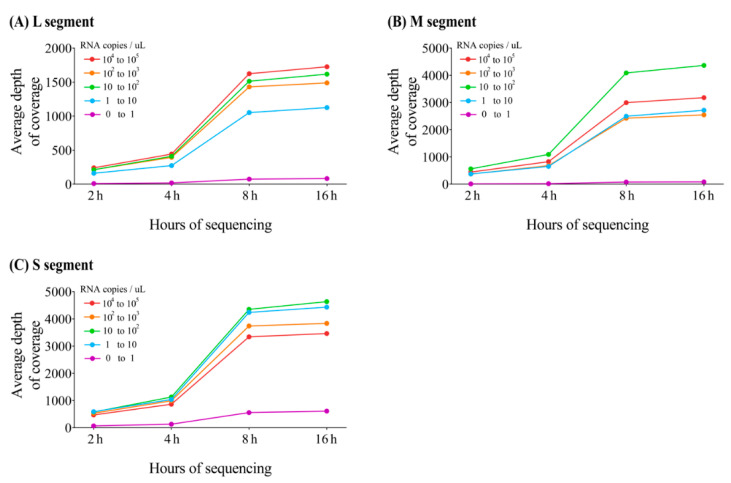
Average depth of coverage for Hantaan virus (HTNV) obtained from MinION sequencing at different cumulated running times. The illustration shows the average depth of coverage generated from amplicon-based MinION sequencing based on (**A**) HTNV L segment, (**B**) M segment, and (**C**) S segment genomes by using different cumulated running times (2, 4, 8, and 16 h).

In the MinION sequencing, 12 nearly whole-genome sequences of HTNV were obtained from the lung tissues of *A. agrarius* captured from Gyeonggi and Gangwon Provinces, ROK. The average depth of coverage for HTNV genomes was calculated using the mapped reads corresponding to sequencing running times (2, 4, 8, and 16 h; Figure 3). After 8 h, MinION sequencing produced nearly complete coverage of the HTNV tripartite genomes with over 1000× average read depth for each segment. The sequencing of Aa19-39 showed a low depth of coverage for the HTNV L, M, and S segments in all the tested specimens owing to the lowest viral load. The genome coverage of HTNV was 65.4–96.8% for the L segment, 90.6–98.5% for the M segment, and 92.1–100% for the S segment (Table 3). The uncovered genome sequences of the HTNV L, M, and S segments were determined using conventional RT-PCR. The 3′ and 5′ terminal sequences were empirically replaced owing to the conserved region of the family *Hantaviridae*. Raw reads obtained after 8 h of sequencing on the MinION platform were extracted and evaluated for indel (insertion and deletion) and mismatch sequences (Table 4). The mean number of single base indels in the homopolymer regions was 3.8 for L segment, 4.4 for M segment, and 1 for S segment. Insertion error was not detected in any of the samples. The total accuracy rates of the HTNV genome sequences obtained using the MinION platform compared to those generated by MiSeq were 99.1–99.9% for the L segment, 99.0–99.9% for the M segment, and 98.3–100% for the S segment. Notably, nucleotide mismatch was the most frequent in the three observed error patterns.

All amplified samples were sequenced in the MiSeq platform to confirm the consensus accuracy of the nanopore sequencing. The coverage of the genome sequences of 12 HTNV strains was 98.2–100% for the L segment, 93.8–100% for the M segment, and 93.8–100% for the S segment (Table S3). The average number of mapped reads and the depth of coverage are shown in Table S4. The uncovered sequences of the HTNV tripartite genomes were determined using conventional RT-PCR. The genomic sequences of the 3′ and 5′ ends were empirically filled with the conserved sequence of the family *Hantaviridae*.

### 3.5. Phylogeographic Analysis

**Figure 4:**
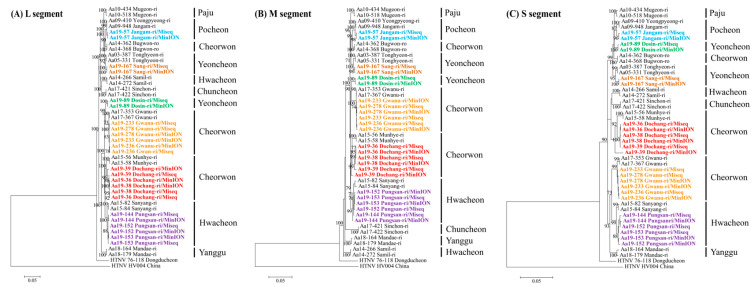
Phylogenetic analysis of Hantaan virus (HTNV) whole-genome sequences by using amplicon next-generation sequencing (NGS) and MiSeq and MinION platforms. Phylogenetic trees were generated using maximum-likelihood method and bootstrap of 1000 iterations based on (**A**) HTNV L segment (1–6533 nt), (**B**) HTNV M segment (1–3616 nt), and (**C**) HTNV S segment (1–1696 nt). The numbers at each node are bootstrap probabilities, as determined for 1000 iterations. The newly obtained HTNV are shown in bold lettering and designated by color indicating the specific sites: blue, Jangam-ri of Pocheon; green, Dosin-ri of Yeoncheon; brown, Sang-ri of Yeoncheon; orange, Gwanu-ri of Cheorwon; red, Dochang-ri of Cheorwon; and violet, Pungsan-ri of Hwacheon. The newly acquired HTNV genomic sequences in this study are described in Table S1. The following other HTNV genome sequences were used in the analysis: HTNV Aa03-387 (L segment, KT934958; M segment, KT934992; S segment, KT935026), Aa05-331 (L segment, KT934962; M segment, KT934996; S segment, KT935030), Aa09-410 (L segment, KU207177; M segment, KU207185; S segment, KU207193), Aa09-948 (L segment, KT934966; M segment, KT935000; S segment, KT935034), Aa10-434 (L segment, KT934970; M segment, KT935004; S segment, KT935038), Aa10-518 (L segment, KT934971; M segment, KT935005; S segment, KT935039), Aa14-266 (L segment, KT934979; M segment, KT935013; S segment, KT935047), Aa14-272 (L segment, KT934980; M segment, KT935014; S segment, KT935048), Aa14-362 (L segment, KT934981; M segment, KT935015; S segment, KT935049), Aa14-368 (L segment, KT934982; M segment, KT935016; S segment, KT935050), Aa15-56 (L segment, KU207179; M segment, KU207187; S segment, KU207195), Aa15-58 (L segment, KU207180; M segment, KU207188; S segment, KU207196), Aa15-82 (L segment, MT012572; M segment, MT012560; S segment, MT012548), Aa15-84 (L segment, MT012573; M segment, MT012561; S segment, MT012549), Aa17-353 (L segment, MT012575; M segment, MT012563; S segment, MT012551), Aa17-367 (L segment, MT012576; M segment, MT012564; S segment, MT012552), Aa17-421 (L segment, MT012577; M segment, MT012565; S segment, MT012553), Aa17-422 (L segment, MT012578; M segment, MT012566; S segment, MT012554), Aa18-164 (L segment, MT012579; M segment, MT012567; S segment, MT012555), Aa18-179 (L segment, MT012580; M segment, MT012568; S segment, MT012556), HTNV 76-118 (L segment, NC_005222; M segment, M14627; S segment, M14626), HV004 (L segment, JQ083393; M segment, JQ083394; S segment, JQ093395).

A total of 12 full-length genomic sequences of HTNV were obtained from multiple regions of Gyeonggi and Gangwon Provinces, ROK. Seven whole-genome sequences of HTNV were recovered from new trapping sites of HFRS endemic areas, including Yeoncheon (Sang-ri), Cheorwon (Dochang-ri), and Hwacheon (Pungsan-ri). The phylogenies of the HTNV L, M, and S segments from Aa19-36, Aa19-38, and Aa19-39 in Cheorwon (Dochang-ri) formed a genetic cluster with the strains collected from Cheorwon (Munhye-ri) (Figure 4). The phylogenetic pattern of the HTNV tripartite genomes from Aa19-57 shared a common ancestor with the HTNV collected in Pocheon. Phylogenetically, the L and M segments of HTNV from Aa19-89 clustered with the HTNV from Cheorwon (Gwanu-ri), whereas the S segment was grouped with the HTNV from Pocheon. The tripartite segments of Aa19-144, Aa19-152, and Aa19-153 in Hwacheon (Pungsan-ri) showed a genetic lineage with HTNV in Hwacheon (Sanyang-ri). The L, M, and S segments of HTNV from Aa19-167 in Yeoncheon (Sang-ri) were phylogenetically grouped with HTNV in Yeoncheon (Tonghyeon-ri). The tripartite HTNV genomes of Aa19-233, Aa19-236, and Aa19-278 from Cheorwon (Gwanu-ri) formed a homologous genetic group with HTNV in Gwanu-ri, Cheorwon.

## 4. Discussion

Rodent-borne orthohantaviruses pose a critical public health threat worldwide [32]. In the ROK, approximately 400 HFRS cases are reported annually, with a mean case fatality rate of 1–4% [33]. HFRS is highly endemic in Gyeonggi and Gangwon Provinces, ROK, and affects both military personnel and civilians [29,34,35,36]. Phylogenetic analyses of hantaviruses delineated a clear molecular epidemiological relationship between HFRS patients and their reservoir hosts, indicating the most putative site of infection [37]. In 2005, HTNV genomic sequences obtained from four US soldier patients diagnosed with HFRS in the ROK showed a phylogenetic link with the viral sequences acquired by targeted rodent trapping in military training locations where the patients had exercised. In our previous studies, multiplex PCR-based NGS was used to obtain nearly whole-genome sequences of HTNV from four ROK and US soldiers during 2013–2015 [18]. Recently, active surveillance with targeted small mammal trapping in the assumed regions of hantavirus outbreaks associated with HFRS enabled the phylogeographical analyses of HTNV within a short distance (approximately 5 km) to track the precise infectious agents and potential exposed sites [38]. Establishing a database of viral genomic sequences to clarify the epidemiological association of patients with infectious sources at a higher resolution can contribute to the development of control strategies of hantavirus outbreaks to prevent endemic transmission [39]. In this study, 12 whole-genome sequences of HTNV newly recovered from *A. agrarius* collected from Gyeonggi and Gangwon Provinces enhanced the resolution of the phylogeographic map of orthohantaviruses. The phylogeny of the HTNV tripartite genomes showed well-supported phylogeographic lineages and genetic diversity in the restricted regions. Furthermore, seven full-length genomic sequences of HTNV were acquired in new trapping sites, including Yeoncheon (Sang-ri), Cheorwon (Dochang-ri), and Hwacheon (Pungsan-ri). Our findings highlight that disease risk assessment and vigilance should be conducted to prevent potential hantavirus outbreaks to humans in HFRS endemic areas.

High-throughput sequencing technology offers a robust platform to investigate the genetic diversity across viral populations, which is essential for understanding viral evolution and pathogenesis [40,41,42,43]. The accuracy of consensus sequences depends on the background error profiling of sequencing technology and the required level of sensitivity [44]. A high level of sequencing coverage is required for population level characterization [45]. Wang et al. revealed that the identification of minor variants at the virus population level required a minimum depth of 400× and 1000× coverage to recognize variants found at a 1% and 0.5% SNP frequency with 99.999% confidence owing to the polymerase-based error and need for amplification, respectively [46]. The targeted amplification of the viral genomes is necessary to achieve these requirements. Three distinct approaches, including SISPA, RNA access, and amplicon-based methods, have been reported for whole-genome sequencing of HTNV from patient and rodent specimens [18,19,20]. Of these methods, amplicon-based NGS demonstrated the most sensitive approach to attain nearly complete genome coverage of HTNV and provided the most read depth by using only 10^2^ viral RNA copies/µL from the lung tissues of wild reservoir rodents [20]. In this study, amplicon-based nanopore sequencing showed nearly full genome coverage of HTNV except for Aa19-39, with an average depth of 1321× for the L segment; 3029× for the M segment; and 3766× for the S segment, after 8 h of sequencing. At the consensus level, the mean accuracy of the HTNV genome sequences using the MinION platform demonstrated 99.8 (L and M segments) and 99.7% (S segment) identities compared to those using Illumina MiSeq. Our data suggest that the amplicon-based method is the most suitable for obtaining high-quality whole-genome sequences of HTNV in the nanopore platform.

The accuracy of genome sequences generated from raw reads plays an important role in metadata analysis for epidemiological surveillance [47]. Nanopore technology provides low-quality reads compared to the Illumina MiSeq platform, which offers 90% of bases with a basecalling accuracy higher than a Q-score of 30 (99.9%) [48]. Currently, the MinION device has error rates of approximately 15% in single reads by using the flow cell (R9 version) [49,50,51,52,53]. Nanopore-based sequencing led to the generation of indel errors that cannot be resolved naturally in homopolymer regions, even with high read depth and coverage [52,54,55]. Single nucleotide indels in the coding regions might induce frameshift mutations. The indel errors in homopolymer sites are a significant limitation to the reliability of sequence accuracy in nanopore technologies. In this study, manual correction of errors at homopolymer sites was performed in the ORF regions to obtain the highest quality consensus sequences of HTNV tripartite genomes. The numbers of nucleotide deletions in HTNV genomes were 2–5 for L segment, 2–6 for M segment, and 1 for S segment. The proteins generated from each segment of HTNV genome are essential for viral function [56,57]. The likelihood of a frameshift mutation naturally occurring in the coding regions of proteins in a viable virus is extremely low. Given these observations, we suggest that manual correction should be appropriately performed in homopolymer sites to resolve indel errors within the coding regions of the HTNV tripartite genomes.

The rapid and accurate generation of full-length viral sequence data is important for improving the approaches to clinical diagnosis (including point-of-care diagnostics), patient-stratified management, epidemiological surveillance, and long-term development of cure strategies [58]. Although the Illumina MiSeq platforms can produce massive and high-quality data, their application for clinical diagnosis and public health has been limited by sequencer size, slowness, and library preparation complexity [59]. In contrast, the MinION device, a compact portable sequencer, offers the application of real-time sequencing in point-of-care tests for infectious diseases, including identification of CHIKV, EBOV, and ZIKV from clinical blood samples [26,59,60]. The major advantages of the MinION system are the reasonable price, the ability to produce real-time data, and portability, which would make it an appropriate device to be used in a hospital or field for rapid diagnosis [24]. In previous studies, a genomic surveillance system based on phylogenetic analyses provided an effective prevention strategy against emerging orthohantavirus outbreaks for mitigating HFRS incidences, such as the restriction of field activities and environmental cleaning to eliminate rodents in endemic areas [37,39]. These surveillance systems depend on the rapid and accurate sequencing of infectious agents from clinical samples. In this study, we showed that amplicon-based nanopore sequencing provides nearly whole-genome sequences for the identification of the phylogeographic diversity of HTNV within 8 h of sequencing. Our study indicates that amplicon-based sequencing with the MinION sequencer can offer the potential for quicker diagnosis and facilitation of rapid decision making in point-of-care testing of HFRS patients in clinical or field situations.

In conclusion, we report 12 complete HTNV sequences newly obtained from *A. agrarius* collected from HFRS-endemic areas of Gyeonggi and Gangwon Provinces, ROK. These results improve the phylogeographic maps for monitoring and responding to hantaviral outbreaks in endemic regions. We found that amplicon-based nanopore sequencing provides rapid and accurate genomic sequences of HTNV for identifying infectious sources and their genetic diversity at the strain level. Our findings suggest the potential for the application of MinION sequencing for the rapid analyses of HTNV from clinical and animal specimens in fields during an endemic outbreak. This study provides significant insights into nanopore sequencing for genomic-based diagnosis and control strategies for hantavirus outbreaks in highly HFRS-endemic areas.

## Figures and Tables

**Table 1:** Trapping results of small mammals collected from Gyeonggi and Gangwon Provinces in 2019.

Species	Small Mammal Trapping Location							Total (%)
	Paju	Pocheon	Pyeongtaek	Yeoncheon	Cheorwon	Chuncheon	Hwacheon	
*Apodemus agrarius*	7	18	41	48	105	38	46	303 (86.6)
*Crocidura lasiura*	-^a^	-	-	1	7	4	1	13 (3.7)
*Crocidura shantungensis*	-	-	-	-	1	1	1	3 (0.9)
*Mus musculus*	-	-	7	-	9	-	1	17 (4.9)
*Myodes regulus*	2	-	-	1	1	-	-	4 (1.1)
*Micromys minutus*	-	-	2	-	1	2	-	5 (1.4)
*Microtus fortis*	-	-	-	-	1	-	-	1 (0.3)
*Rattus norvegicus*	-	-	3	-	-	-	-	3 (0.9)
*Tscherskia triton*	-	-	-	-	-	-	1	1 (0.3)
Total	9	18	53	50	125	45	50	350 (100)

^a^: no collection.

**Table 2:** Serological and molecular prevalence of Hantaan virus (HTNV) in *Apodemus agrarius* captured from Gyeonggi and Gangwon Provinces in 2019.

Trapping Site	Number of Captured *A. agrarius*	Seropositivity for Anti-HTNV IgG (%)			HTNV RNA Positivity (%)		
		Male	Female	Total	Male	Female	Total
Paju	7	0/2	0/5	0/7	-^a^	-	-
Pocheon	18	1/9 (11.1)	0/9	1/18 (5.6)	1/1 (100)	-	1/1 (100)
Pyeongtaek	41	0/23	0/18	0/41	-	-	-
Yeoncheon	48	2/22 (9.1)	3/26 (11.5)	5/48 (10.4)	1/2 (50)	1/3 (33.3)	2/5 (40)
Cheorwon	105	5/60 (8.3)	2/45 (4.4)	7/105 (6.7)	4/5 (80)	2/2 (100)	6/7 (85.7)
Chuncheon	38	0/18	1/20 (5)	1/38 (2.6)	-	0/1	0/1
Hwacheon	46	4/20 (20)	2/26 (7.7)	6/46 (13.0)	3/4 (75)	0/2	3/6 (50)
Total	303	12/154 (7.8)	8/149 (5.4)	20/303 (6.6)	9/12 (75)	3/8 (37.5)	12/20 (60)

^a^: seronegative sample was not analyzed using RT-PCR.

**Table 3:** Results of MinION sequencing based on viral RNA copy number of Hantaan virus (HTNV) in the lung tissues of rodents collected from Gyeonggi and Gangwon Provinces, ROK, in 2019.

Viral RNA Copy Number (Copies/µL)	Sample	Site	Anti-HTNV IgG Titer	Nested RT-PCR	*C*t Value	HTNV Genomes, % Coverage (Minimum Read Depth, >10×)		
						L Segment	M Segment	S Segment
10^4^ to 10^5^	Aa19-233	Cheorwon	1:64 ^a^	Pos	20.5	96.8	98.5	100
	Aa19-278	Cheorwon	1:32 ^a^	Pos	22.2	92.9	98.4	100
10^2^ to 10^3^	Aa19-236	Cheorwon	1:32 ^a^	Pos	29.4	95.3	98.4	100
	Aa19-36	Cheorwon	1:512 ^b^	Pos	29.7	91.7	98.4	100
10 to 10^2^	Aa19-89	Yeoncheon	1:64 ^a^	Pos	31.8	95.9	98.4	100
	Aa19-57	Pocheon	1:8096 ^b^	Pos	32.7	95.8	98.4	100
	Aa19-167	Yeoncheon	1:256 ^a^	Pos	34.0	95.9	96.4	100
	Aa19-38	Cheorwon	1:16,384 ^b^	Pos	34.4	92.3	98.4	100
	Aa19-153	Hwacheon	1:4096 ^b^	Pos	34.5	92.7	98.4	100
1 to 10	Aa19-152	Hwacheon	1:512 ^a^	Pos	36.4	82.6	97.0	92.1
	Aa19-144	Hwacheon	1:256 ^b^	Pos	37.3	88.8	96.4	95.1
0 to 1	Aa19-39	Cheorwon	1:512 ^b^	Pos	40.0	65.4	90.6	92.2

HTNV: Hantaan virus; IgG, immunoglobulin G; nested RT-PCR, nested reverse transcription-polymerase chain reaction; Ct, cycle threshold; Aa, *Apodemus agrarius*; Pos, Positive; Neg, Negative; ^a^, IFA test was performed from heart fluids; ^b^, IFA test was performed from sera.

**Table 4:** Sequencing error and consensus accuracy of amplicon-based MinION sequencing for Hantaan virus.

Viral RNA Copy Number (Copies/µL)	Sample	Site	Ct Value	L Segment				M Segment				S Segment			
				Insertion	Deletion	Mismatch	Accuracy ^a^	Insertion	Deletion	Mismatch	Accuracy	Insertion	Deletion	Mismatch	Accuracy
10^4^ to 10^5^	Aa19-233	Cheorwon	20.5	0	3	12	99.8	0	5	2	99.9	0	1	4	99.8
	Aa19-278	Cheorwon	22.2	0	4	9	99.9	0	6	2	99.9	0	1	0	100
10^2^ to 10^3^	Aa19-236	Cheorwon	29.4	0	5	9	99.9	0	6	3	99.9	0	1	1	99.9
	Aa19-36	Cheorwon	29.7	0	3	12	99.8	0	5	2	99.9	0	1	1	99.9
10 to 10^2^	Aa19-89	Yeoncheon	31.8	0	3	6	99.9	0	5	6	99.8	0	1	1	99.9
	Aa19-57	Pocheon	32.7	0	4	9	99.9	0	5	4	99.9	0	1	2	99.9
	Aa19-167	Yeoncheon	34.0	0	4	8	99.9	0	4	4	99.9	0	1	0	100
	Aa19-38	Cheorwon	34.4	0	3	12	99.8	0	5	7	99.8	0	1	2	99.9
	Aa19-153	Hwacheon	34.5	0	5	14	99.8	0	4	3	99.9	0	1	3	99.8
1 to 10	Aa19-152	Hwacheon	36.4	0	5	18	99.7	0	2	11	99.7	0	1	7	99.6
	Aa19-144	Hwacheon	37.3	0	4	14	99.8	0	4	11	99.7	0	1	7	99.6
0 to 1	Aa19-39	Cheorwon	40.0	0	2	59	99.1	0	2	36	99.0	0	1	28	98.3
Total		Average (%)		0	3.8	15.2	99.8	0	4.4	7.6	99.8	0	1	4.7	99.7

*C*t, cycle threshold; Aa, *Apodemus agrarius*; ^a^, Percentage of nucleotide similarity between Oxford nanopore and Illumina MiSeq platforms.

## Data Availability

All the data generated for this publication have been included in the current manuscript.

## Supplementary Materials

The following are available online at https://www.mdpi.com/article/10.3390/v13050847/s1. Figure S1, Phylogenetic analysis based on the mitochondrial cytochrome *b* gene of *Apodemus* spp; Table S1, GenBank accession numbers of Hantaan virus sequences obtained in this study; Table S2, Topography and GPS coordinates of the trapping sites; Table S3, Results of MiSeq sequencing from rodent lung tissues by viral RNA copy number of Hantaan virus (HTNV) collected in Gyeonggi and Gangwon Provinces, ROK, 2019; Table S4, Summary of mapped reads and depth of coverages for Hantaan virus by amplicon-based NGS in MiSeq platform..
