# Supplementary material for: Multiplex PCR-Based Nanopore Sequencing and Epidemiological Surveillance of Hantaan orthohantavirus in Apodemus agrarius, Republic of Korea
Source: Viruses. 2021 May 6;13(5):847. doi: 10.3390/v13050847 (PMC8148566; doi:10.3390/v13050847)
Supplement: Supplementary file 1 [file viruses-13-00847-s001.zip › supplementary Table.pdf]

Table S1. GenBank accession numbers of Hantaan virus sequences obtained in this study.

Sample	MiSeq platform			MinION platform		
	L	M	S	L	M	S
Aa19-36	MW796122	MW796134	MW796146	MW796158	MW796170	MW796182
Aa19-38	MW796123	MW796135	MW796147	MW796159	MW796171	MW796183
Aa19-39	MW796124	MW796136	MW796148	MW796160	MW796172	MW796184
Aa19-57	MW796125	MW796137	MW796149	MW796161	MW796173	MW796185
Aa19-89	MW796126	MW796138	MW796150	MW796162	MW796174	MW796186
Aa19-144	MW796127	MW796139	MW796151	MW796163	MW796175	MW796187
Aa19-152	MW796128	MW796140	MW796152	MW796164	MW796176	MW796188
Aa19-153	MW796129	MW796141	MW796153	MW796165	MW796177	MW796189
Aa19-167	MW796130	MW796142	MW796154	MW796166	MW796178	MW796190
Aa19-233	MW796131	MW796143	MW796155	MW796167	MW796179	MW796191
Aa19-236	MW796132	MW796144	MW796156	MW796168	MW796180	MW796192
Aa19-278	MW796133	MW796145	MW796157	MW796169	MW796181	MW796193

Table S2. Topography and GPS coordinates of the trapping sites.

Trapping site			Topography	GPS coordinates
Gyeonggi Province	Paju	Jeogam-ri	fields, hills, and mountains	37°58′52.1″N 126°59′49.3″E
	Pocheon	Jangam-ri	fields, unmanaged grasses, and mountains	38°3′40″N 127°21′42″E
		Jail-ri	fields, unmanaged grasses, and herbaceous vegetations	38°6′51″N 127°17′58″E
	Pyeongtaek	Jeokbong-ri	sloping hillsides, herbaceous vegetations, and forests	37°4′46.21″N 127°2′15.65″E
		Pyeongseong-ri	fields, unmanaged grasses, and herbaceous vegetations	36°57′35″N 127°3′39″E
	Yeoncheon	Hyunga-ri	herbaceous vegetations and farmlands	38°7′2.9″N 127°4′27″E
		Dongi-ri	fields, unmanaged grasses, and herbaceous vegetations	38°1′3″N 126°59′31″E
		Ohkye-ri	mountains and forests	38°7′25″N 127°4′5″E
		Sang-ri	mountains and forests	38°8′4″N 127°3′37″E
		Yangwon-ri	fields, unmanaged grasses, and herbaceous vegetations	37°59′47″N 127°2′3″E
		Bugok-ri	fields, unmanaged grasses, and herbaceous vegetations	38°3′52″N 127°8′58″E
		Dosin-ri	fields, unmanaged grasses, and herbaceous vegetations	38°9′31″N 127°5′51″E
Gangwon Province	Cheorwon	Eupnae-ri	fields, unmanaged grasses, and herbaceous vegetations	38°18′00″N 127°27′33″E
		Munhye-ri	herbaceous vegetations and farmlands	38°11′18″N 127°22′2″E
		Dochang-ri	fields, hills, forests, and mountains	38°15′27″N 127°21′25″E
		Chungyang-ri	farmlands and mountains	38°15′2″N 127°21′56″E
		Gwanu-ri	farmlands and herbaceous vegetations	38°14′15.4″N 127°13′35.2″E
	Chuncheon	Gamjeong-ri	farmlands, herbaceous vegetations, and mountains	37°54′4″N 127°48′27″E
		Sinchon-ri	farmlands, herbaceous vegetations, and mountains	37°51′10″N 127°47′32″E
		Jinae-ri	fields, hills, and mountains	37°57′45.8″N 127°42′54.4″E
		Balsan-ri	farmlands, unmanaged grasses, and herbaceous vegetations	37°57′45.8″N 127°45′4.5″E
		Yulmun-ri	hills and herbaceous vegetations	37°55′32.2″N 127°44′34.8″E
	Hwacheon	Pyungsan-ri	fields, forests, and mountains	38°8′28″N 127°44′15″E

Table S3. Results of MiSeq sequencing from rodent lung tissues by viral RNA copy number of Hantaan virus (HTNV) collected in Gyeonggi and Gangwon Provinces, ROK, 2019.

Viral RNA copy number (Copies/μL)	Sample	Site	Anti-HTNV IgG titer	Nested RT-PCR	Ct value	HTNV genomes, % coverage		
						L segment	M segment	S segment
10^4 to 10^5	Aa19-233	Cheorwon	1:64[a]	Pos	20.5	100	100	100
	Aa19-278	Cheorwon	1:32[a]	Pos	22.2	100	100	100
10^2 to 10^3	Aa19-236	Cheorwon	1:32[a]	Pos	29.4	100	100	100
	Aa19-36	Cheorwon	1:512[b]	Pos	29.7	96.9	100	100
10 to 10^2	Aa19-89	Yeoncheon	1:64[a]	Pos	31.8	98.9	98.5	100
	Aa19-57	Pocheon	1:8,096[b]	Pos	32.7	98.9	98.5	100
	Aa19-167	Yeoncheon	1:256[a]	Pos	34.0	100	98.6	100
	Aa19-38	Cheorwon	1:16,384[b]	Pos	34.4	98.2	98.3	100
	Aa19-153	Hwacheon	1:4,096[b]	Pos	34.5	100	98.5	100
1 to 10	Aa19-152	Hwacheon	1:512[a]	Pos	36.4	95.6	98.3	100
	Aa19-144	Hwacheon	1:256[b]	Pos	37.3	94.5	93.8	100
0 to 1	Aa19-39	Cheorwon	1:512[b]	Pos	40.0	92.3	94.1	100

HTNV, Hantaan virus; IgG, immunoglobulin G; nested RT-PCR, nested reverse transcription-polymerase chain reaction; Ct, cycle threshold; Aa, *Apodemus agrarius*; Pos, Positive; Neg, N egative; [a]; IFA test was performed from heart fluids; [b]; IFA test was performed from sera.

Table S4. Summary of mapped reads and depth of coverages for Hantaan virus by amplicon-based NGS in MiSeq platform.

Viral RNA copy number (Copies/μL)	Sample	Total reads	Reads mapped to reference/Total reads (%)	L segment		M segment		S segment	
				Reads mapped to reference (%)	Depth of coverage	Reads mapped to reference (%)	Depth of coverage	Reads mapped to reference (%)	Depth of coverage
10^4 to 10^5	Aa19-233	1,630,766	1,575,693 (96.6)	518,542 (31.8)	9,687.1	614,309 (37.7)	20,566.1	442,842 (27.2)	31,539.4
	Aa19-278	1,381,944	1,343,293 (97.2)	537,253 (38.9)	9,976.4	549,724 (39.8)	18,126.3	256,316 (18.5)	18,234.5
10^2 to 10^3	Aa19-236	2,578,890	2,325,129 (90.2)	784,074 (30.4)	14,644.8	750,025 (29.1)	25,143.6	791,030 (30.7)	56,569.0
	Aa19-36	290,576	284,457 (97.9)	100,494 (34.6)	1,880.4	103,340 (35.6)	3,496.8	80,623 (27.7)	5,790.1
10 to 10^2	Aa19-89	210,878	206,764 (98.0)	73,586 (34.9)	1,377.1	76,690 (36.4)	2,588.3	56,488 (26.8)	4,031.3
	Aa19-57	323,856	315,500 (97.4)	100,682 (31.1)	1,880.9	123,168 (38.0)	4,128.8	91,650 (28.3)	6,517.6
	Aa19-167	1,176,050	1,131,359 (96.2)	379,829 (32.3)	7,113.7	403,430 (34.3)	13,581.6	348,100 (29.6)	24,985.6
	Aa19-38	308,620	302,858 (98.1)	95,107 (30.8)	1,783.9	117,222 (38.0)	3,965.8	90,529 (29.3)	6,467.0
	Aa19-153	278,190	267,345 (96.1)	87,679 (31.5)	1,630.0	105,837 (38.0)	3,542.9	73,829 (26.5)	5,165.2
1 to 10	Aa19-152	441,564	415,477 (94.1)	105,904 (24.0)	1,969.6	153,056 (34.7)	5,067.1	156,517 (35.4)	10,737.0
	Aa19-144	310,472	245,605 (79.1)	85,996 (27.7)	1,572.9	78,116 (25.2)	2,500.6	81,493 (26.2)	5,793.7
0 to 1	Aa19-39	629,654	386,490 (61.4)	54,677 (8.7)	972.8	189,412 (30.1)	5,864.4	142,401 (22.6)	9,861.3
**Total**	**Average (%)**	**796,788**	**733,330.8 (92.0)**	**243,651.9 (30.6)**	**4,540.8**	**272,027.4 (34.1)**	**9,047.7**	**217,651.5 (27.3)**	**15,474.3**

Aa, *Apodemus agrarius*; C_t, cycle threshold; L, large; M, medium; S, small.

The depth of coverage was determined by the number of mapped reads (read length × number of reads matching the reference/reference genome size).
