# Supplementary figures and images for: Multiplex PCR-Based Nanopore Sequencing and Epidemiological Surveillance of Hantaan orthohantavirus in Apodemus agrarius, Republic of Korea
Source: Viruses. 2021 May 6;13(5):847. doi: 10.3390/v13050847 (PMC8148566; doi:10.3390/v13050847)

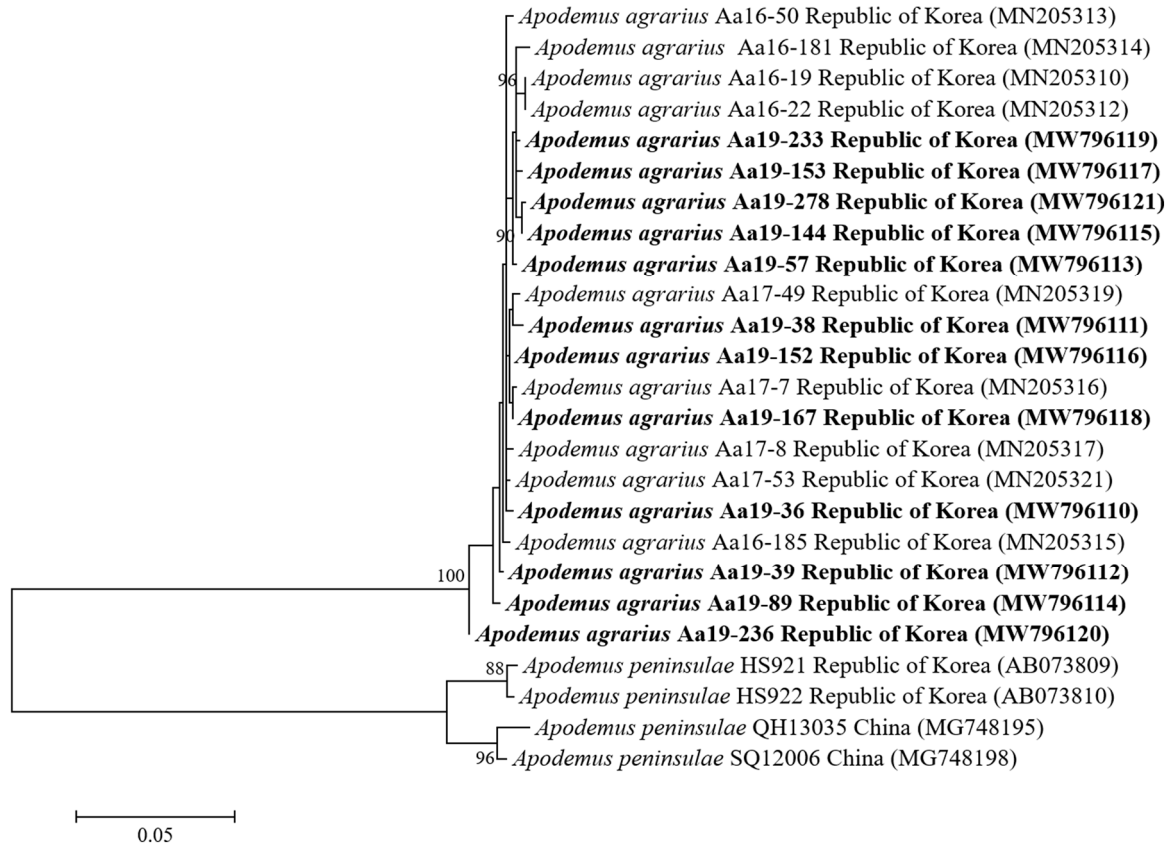

**Figure S1.** Phylogenetic analysis based on the mitochondrial cytochrome *b* gene of *Apodemus* spp.

Supplement: Supplementary file 1 [file viruses-13-00847-s001.zip › S_Fig1.pdf]
